# Peritoneal dialysis in Qatar: A model of innovation and excellence

**DOI:** 10.5339/qmj.2025.31

**Published:** 2025-06-11

**Authors:** Mohammed Ezzat, Ashraf Fawzy, Mohamad M. Alkadi, Hassan Al-Malki, Abdullah I. Hamad

**Affiliations:** 1 Division of Nephrology, Department of Medicine, Hamad Medical Corporation, Doha, Qatar *Email: malkadi@hamad.qa

Peritoneal dialysis (PD) is one of the three primary renal replacement therapies (RRT) for patients with end-stage kidney disease (ESKD), alongside hemodialysis (HD) and kidney transplantation. PD was first introduced in Qatar in 1976 for the management of acute kidney injury during hospitalization. In 1997, PD was expanded as a home-based RRT for patients with ESKD, offering both continuous ambulatory PD and automated PD. This provided an alternative to in-center HD, supporting greater autonomy, flexibility, and quality of life.^1,3^

Hamad Medical Corporation, Qatar’s main public healthcare provider, remains the sole provider of PD services in the country. The national PD program has steadily expanded and currently serves approximately 180 patients, representing ~13% of the national dialysis population.^2,3^ This growth reflects a comprehensive patient-centered approach that integrates high-quality clinical care, structured education, and efficient coordination of dialysis access. The continuous adoption of modern technologies and the evolution of treatment strategies have yielded favorable outcomes. Notably, the program has outperformed some Western benchmarks, driven by a patient-centered philosophy and a commitment to continuous professional development.^4,5^

## KEY INNOVATIONS AND STRATEGIC ENHANCEMENTS

### Incremental peritoneal dialysis (iPD)

In 2018, we implemented a structured iPD protocol, initiating patients on 1–2 daily exchanges and progressively intensifying therapy based on clinical symptoms, residual urine output, and dialysis adequacy targets. This individualized approach helped preserve residual kidney function, maintain fluid and solute balance, improve treatment adaptation, and reduce both early dropout rates and hospitalizations. These outcomes align with international data and the International Society for Peritoneal Dialysis (ISPD) guidelines, which support iPD as standard practice for eligible patients.^6,7^

### Improving access to medications

Financial limitations had previously restricted access to key medications for ESKD, particularly those addressing mineral and bone disease and anemia. In 2019, a financial support program was introduced, initially targeting individuals with mineral and bone diseases. This led to an increase in the proportion of patients achieving ISPD phosphorus targets from 55% to 81%, and parathyroid hormone targets from 52% to 65%. In 2023, the program expanded to anemia management, increasing the proportion of patients meeting hemoglobin targets from 62% to 72%. These improvements highlight the positive impact of improving access to treatment on key clinical indicators.

### Managing persistent hypokalemia

Persistent hypokalemia affected approximately one-third of the PD population, often requiring poorly tolerated potassium supplements. In 2019, a multidisciplinary protocol was introduced, focusing on magnesium correction, dietary counseling, and the use of low-dose spironolactone in eligible patients. This approach effectively resolved hypokalemia in most cases and reduced reliance on oral supplements, with no serious adverse events reported.^3^

### COVID-19 response and remote care model

During the COVID-19 pandemic, and in alignment with national infection control measures, routine PD clinic visits were transitioned to telephone consultations, with in-person visits reserved for urgent concerns such as fluid overload, suspected peritonitis, or catheter dysfunction. This model facilitated treatment adherence, enabled early detection of complications, and minimized exposure risk. Patient feedback indicated high satisfaction with remote care, and unplanned hospital visits remained low. These results underscore the PD program’s adaptability and resilience during public health crises.

### Hybrid dialysis (HyD)

The HyD model was introduced in 2020 to address challenges such as inadequate solute clearance or fluid overload in PD patients. It combines 6 days of PD with one weekly HD session, optimizing solute removal and fluid balance without compromising patient autonomy. Among 23 enrolled patients, HyD led to improvements in blood pressure control, biochemical and nutritional status, reduced emergency department visits, and higher patient satisfaction.^8^ These outcomes align with global data supporting HyD as a safe and effective strategy for patients with declining peritoneal membrane function.

### Reducing culture-negative peritonitis

In 2021, the rate of culture-negative peritonitis was twice the ISPD benchmark, often leading to unnecessary empiric antibiotic use and catheter removals. A comprehensive diagnostic protocol was introduced, incorporating dual sample submission (standard and pediatric blood culture bottles) and close coordination with the microbiology laboratory for 16S rRNA sequencing and expanded testing for fungal and mycobacterial infections. This initiative significantly reduced culture-negative peritonitis rates and unnecessary catheter removals, while improving pathogen identification.^9,10^

### Urgent-start PD pathway

PD dropout has posed a persistent challenge, with approximately one-third of patients initially planned for PD ultimately transitioning to HD when urgent dialysis was required. Previously, such patients received both a tunneled HD catheter and a PD catheter. However, many subsequently opted to continue with HD and declined PD training. To address this, an urgent start PD pathway was introduced in October 2022, enabling PD initiation within 48 hours of catheter insertion without interim HD. This approach significantly reduced the number of transitions to HD, access-related complications, and the overall healthcare burden.^11^

## CONCLUSION

The PD program in Qatar exemplifies innovation, clinical excellence, and a sustained commitment to patient-centered care. This program has consistently evolved by adopting incremental and HyD models, implementing advanced diagnostics, and addressing social determinants of health to meet patient needs and optimize outcomes. Its agile response during the COVID-19 pandemic further demonstrated resilience and operational effectiveness. Collectively, these efforts offer a practical model for other programs aiming to deliver safe, effective, and sustainable PD care, serving as a roadmap for future advancements in dialysis across the region.

## Conflicts of interest

None.
